# Solving an Old Dogma: Is it an Arteriole or a Venule?

**DOI:** 10.3389/fnagi.2019.00289

**Published:** 2019-10-22

**Authors:** Matthew MacGregor Sharp, Theodore P. Criswell, Howard Dobson, Ciara Finucane, Ajay Verma, Roxana O. Carare

**Affiliations:** ^1^Faculty of Medicine, Institute for Life Sciences, University of Southampton, Southampton, United Kingdom; ^2^inVICRO, Boston, MA, United States; ^3^Biogen Idec, Cambridge, MA, United States

**Keywords:** venules, arterioles, intramural cell, transmission electron microscopy, morphology

## Abstract

There are very few reliable methods in the literature to discern with certainty between cerebral arterioles and venules. Smooth muscle cells (SMC) and pericytes are present in both arterioles and venules, so immunocytochemistry for markers specific to intramural cells (IMC) is unreliable. This study employed transmission electron microscopy (TEM) and a canine brain to produce robust criteria for the correct identification of cerebral arterioles and venules based on lumen:vessel wall area, tested against the less accurate lumen diameter:vessel wall thickness. We first used morphology of IMC to identify two distinct groups of vessels; group 1 with morphology akin to venules and group 2 with morphology akin to arterioles. We then quantitatively assessed these vessels for lumen:vessel wall area ratio and lumen diameter:wall thickness ratio. After assessing 112 vessels, we show two distinct groups of vessels that can be separated using lumen:vessel wall area (group 1, 1.89 −10.96 vs. group 2, 0.27–1.57; *p* < 0.001) but not using lumen diameter:vessel wall thickness where a substantial overlap in ranges between groups occurred (group 1, 1.58–22.66 vs. group 2, 1.40–11.63). We, therefore, conclude that lumen:vessel wall area is a more sensitive and preferred method for distinguishing cerebral arterioles from venules. The significance of this study is wide, as cerebral small vessel disease is a key feature of vascular dementia and understanding the pathogenesis relies on correct identification of vessels.

## Introduction

Throughout the literature on anatomical and pathological features of the cerebral circulation, there is a lack of consistent criteria for differentiating cerebral venules from arterioles. Most studies identify arterioles by ratio of lumen diameter to vessel wall thickness and/or the presence of vascular smooth muscle cells (SMC), either immunohistochemically, such as with α-smooth muscle actin stain, or microscopically by identifying a thick tunica media containing multiple SMC layers (Scharrer, 1940; Skalli et al., 1989; Albargothy et al., 2017). While this method allows for identification of larger leptomeningeal vessels that possess multiple SMC layers, its accuracy for identifying the smaller arterioles and venules found in brain parenchyma is questionable. These smaller vessels, derived from the neural crest of the ectoderm, both possess a form of tunica media containing a single layer of SMC and/or pericytes [both classed as intramural cells (IMC)] embedded within the same endothelial basement membrane (Rhodin, 1968; Roggendorf and Cervós-Navarro, 1983) and so could potentially have very similar ratios of lumen diameter to vessel wall thickness. Cerebral arterioles for example have been shown to be much thinner-walled than their larger counterparts (Rhodin, 1967; Dahl, 1973). Venules also traditionally have thinner walls and larger lumina, although to our knowledge, no study has provided quantification of the respective ratios for each vessel type. To confuse matters further, SMCs and pericytes both express α-smooth muscle actin and share many functional characteristics, particularly at post-capillary level (Rhodin, 1962, 1968, 1980; Krisch et al., 1984; Krisch, 1988; Skalli et al., 1989; Zhang et al., 1990; Owens et al., 2008; Díaz-Flores et al., 2009; Armulik et al., 2011). Several key differences in morphology and distribution have been observed using transmission electron microscopy (TEM) but these have been separately described and not directly compared for each vessel type (see Table 1 for summary of key differences; Rhodin, 1962, 1967, 1968, 1980; Dahl, 1973; Motta, 1990; Armulik et al., 2011).

**Table 1:** Characteristics of the intramural cells found within the walls of intracerebral arterioles and venules, as seen in transverse sections though the vessels with transmission electron microscopy.

Ultrastructural features	Arteriole	Venule
Complete/incomplete layer around vessel	Always complete	Often incomplete
Intramural cell shape	Square/rectangular, “block like”	Flattened/spindle-like
Number of intramural cell layers	Most commonly 1, occasionally 2, rarely 3	1 Layer, occasionally cells overlap
Caveolae (flask shaped invaginations of the cell membrane)	Present	Rare
Electron dense myosin bands (part of contractile apparatus)	Present	Rare

Currently, to correctly distinguish cerebral venules and arterioles a combination of immunohistochemistry for α-smooth muscle actin expression and ultrastructural examination by TEM (particularly for IMC morphology) is required. Several studies claiming to just use ratio of lumen diameter to vessel wall thickness fail to recognize that vessels are rarely smoothly circumscribed or fully circular and that vessel wall thickness can be dramatically altered depending on the plane of section through the tissue (Weller et al., 1998, 2009; Mendel et al., 2013). Ultimately, this has made distinguishing small venules from arterioles challenging and studies have fundamentally confused the two (Scharrer, 1940; Roggendorf et al., 1978; Roggendorf and Cervós-Navarro, 1983). This is particularly relevant to cerebral small vessel disease in which both arterioles and venules can be affected by pathological changes such as arteriolar hyalinosis (collagenosis/fibrosis), fibrinoid necrosis or venous collagenosis. Correct identification of the type of vessel involved may help to facilitate more targeted treatment strategies.

In this study, we use TEM to ascertain if the ratio of lumen to vessel wall area is more accurate than the ratio of lumen diameter to vessel wall thickness in correctly differentiating the small arterioles and venules found in brain parenchyma. We chose to use TEM as the resolution of normal light microscopy does not allow for clear identification of the vessel wall and its components. We first differentiate vessel types based on morphology and arrangement of IMCs from published literature that have utilized TEM (Rhodin, 1962, 1968, 1980; Krisch et al., 1984; Krisch, 1988; Skalli et al., 1989; Zhang et al., 1990; Owens et al., 2008; Díaz-Flores et al., 2009; Armulik et al., 2011). We then analyze these vessels for both ratio of lumen to vessel wall area and ratio of lumen diameter to vessel wall thickness. Image analysis was performed using a beagle dog brain but the TEM methods described here are easily transferrable to any mammalian tissue to produce a robust criterion to differentiate cerebral arterioles from venules.

## Materials and Methods

### Animals

The 12-year-old beagle brain tissue used in this study was supplied and intracardially perfused by the Lawson Health Sciences Research Institute, London, ON, Canada. Euthanasia was performed respecting ethical governance rules at the site.

### Tissue Preparation for Transmission Electron Microscopy

Under routine general anesthesia a 12-year-old Beagle dog was perfused intracardially with 0.1 M piperazine-N, N′-bis(2-ethanesulfonic acid) buffer (PIPES, PH 7.2) followed by 4% formaldehyde plus 3% glutaraldehyde in 0.1 M PIPES buffer at PH 7.2. The brain was removed, post-fixed overnight in fresh fixative and then sectioned into 10 mm coronal slices. 60 μm coronal slices of left frontal, parietal and occipital lobes were cut using a Leica VT1000 vibratome. Representative sections of white matter and cortical gray matter were microdissected and processed for TEM according to our own optimized protocols (Sharp et al., 2017).

Tissue blocks were trimmed and 80 nm ultrathin sections cut using an Ultracut E microtome. The sections were transferred onto copper grids and counter-stained with lead citrate. Grids were examined using a Hitachi H7000 TEM operating a EMSIS MegaView III digital camera and EMSIS image capture software (formerly iTEM software, Universal TEM Imaging platform, Soft Imaging System, Münster, Germany).

### Image Acquisition

Grids were examined in a sequential manner from top left to bottom right. Vessels sectioned in transverse plane were first imaged at a higher magnification for IMC analysis and then lower magnification for lumen:wall ratio analysis. We identified vessels to be in transverse plane if all components of the vessel wall could be clearly resolved.

**Figure 1:**
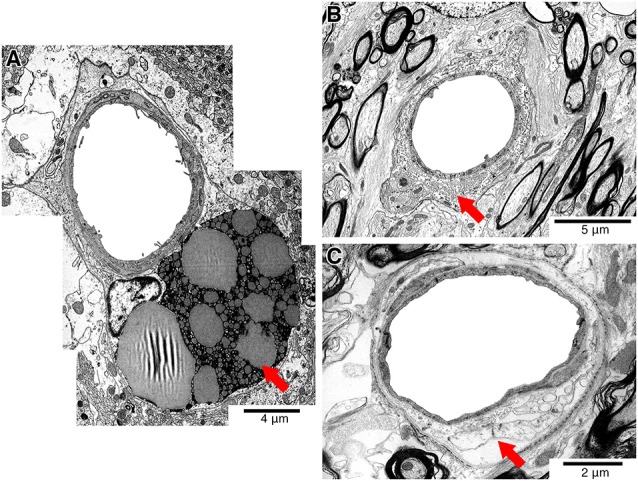
Representative electron micrographs showing pathology that affects the vessel wall (red arrows). Vessels showing perivascular lipofuscin cuffs **(A)**, perivenous collagenosis **(B)** or dilated perivascular spaces **(C)** were excluded from the study.

To avoid imaging capillaries and post-capillary venules, we excluded vessels with a surface area of less than 70 μm^2^. We also did not include vessels that showed artifact or vessel altering pathology such as dilated perivascular spaces, extensive perivascular lipofuscin cuffs or perivenous collagenosis which would often exceed the entire vessel surface area (Black, 2011) (Figure 1).

### Image Analysis

**Figure 2:**
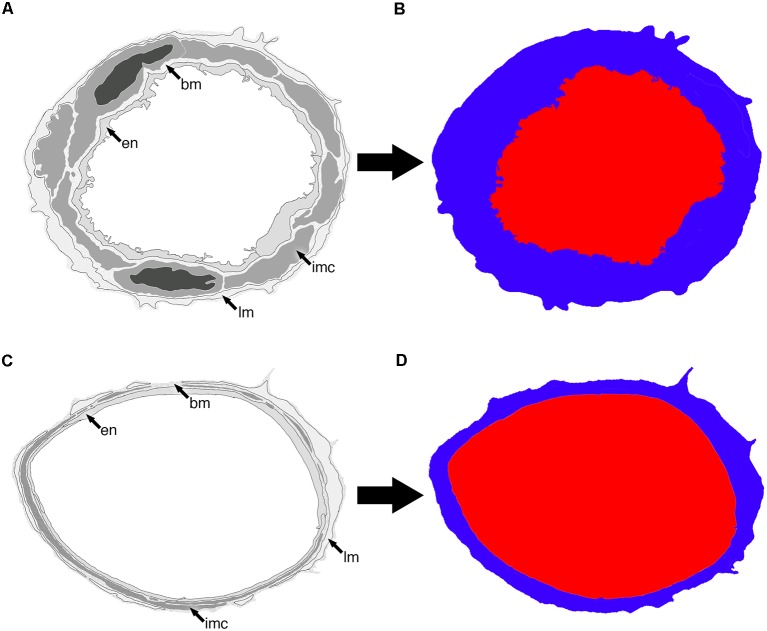
Representative diagram of two vessels **(A,C)** and how they appear once segmented into lumen (red) and vessel wall (blue; **B,D**). The endothelium (en), basement membrane (bm), intramural cells (IMC; sm) and leptomeninges (lm) are all included as part of the vessel wall.

Higher power images of each vessel were used to qualitatively asses the morphology of IMC based on the ultrastructural features listed in Table 1. Low power images of the same vessels were then used to calculate both vessel lumen:wall ratio and ratio of lumen diameter to vessel wall thickness. Briefly, the vessel lumen and vessel wall were outlined in Adobe Photoshop CS6 using two distinct colors, exported as a TIFF file and then imported into ImageJ (National Institutes of Health developed imaging processing program). Image size metrics and an ImageJ threshold function was used to measure both the surface area and width of each demarcated feature to create either a lumen:wall or lumen diameter:wall thickness ratio (Figure 2). Statistical analysis was performed using SPSS and a Mann–Whitney *U*-test (significance set at *P* < 0.05).

## Results

**Figure 3:**
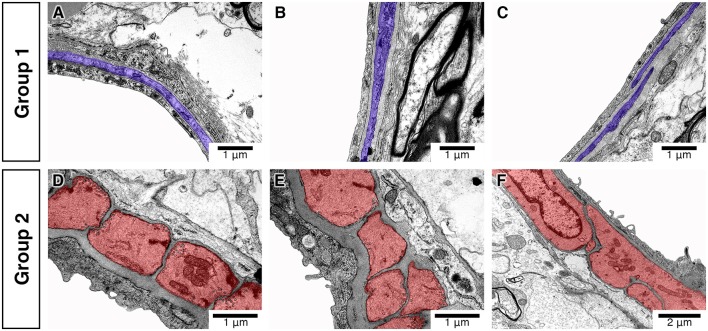
Differences in the morphology and distribution of IMC. Vessels classified into group 1 **(A–C)** have spindle-like IMC (purple) with few caveolae and electron-dense myosin bands. Vessels classified into group 2 **(D–F)** have block-like IMC (red) with multiple caveolae and electron-dense myosin bands.

In total, 112 vessels were analyzed. We first assessed the vessels for the morphology and arrangement of IMCs. We found that the vessels could be separated into two distinct groups that we have labeled group 1 (81 vessels) and group 2 (31 vessels). Group 1 vessels consisted of IMCs that formed a discontinuous layer and appeared spindle-like with few caveolae and electron-dense myosin bands. Group 2 vessels formed a continuous single layer and appeared block-like with multiple caveolae and electron-dense myosin bands (Figure 3). These results agree with previous findings (Table 1).

In group 1 vessels, the area of the lumen was on average 3.9489 times bigger than that of the vessel wall (range 1.89–10.96). The diameter of the lumen was on average 7.3199 times bigger than the thickness of the vessel wall (range 1.58–22.66). The large range of group 1 vessels was partly due to an exponential increase in ratio with the largest vessels analyzed. In group 2 vessels, the area of the lumen was on average 0.9317 times smaller than that of the vessel wall (range 0.27–1.57) but the diameter of the lumen was on average 4.348 times bigger than the thickness of the vessel wall (range 1.40–11.63).

**Figure 4:**
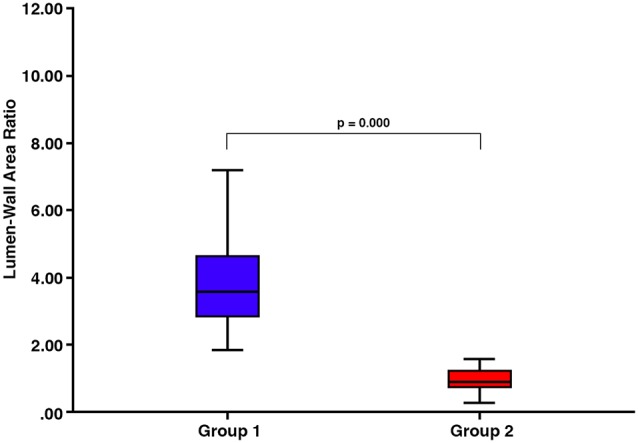
There are significant differences in the lumen:wall area ratio between vessels classified into group 1 and those classified into group 2 (*p* = 0.000).

**Figure 5:**
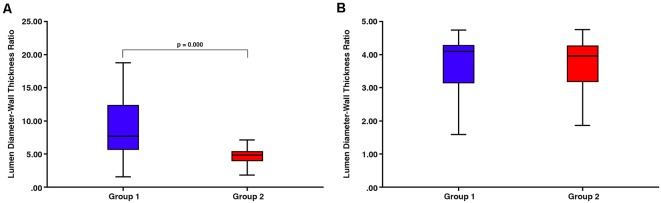
**(A)** There is a substantial overlap in ranges between group 1 and group 2 vessels despite the overall significant difference in the lumen diameter:wall thickness ratio (*p* = 0.000). **(B)** There is no significant difference between any vessels below the average lumen diameter:wall thickness ratio seen in group 2 vessels (4.97, Mann–Whitney test, *p* = 0.817).

There was a significant difference in lumen:wall area ratio between group 1 and group 2 vessels (Mann–Whitney test, *p* = 0.000) with no overlap in the range of lumen:wall area ratio (Figure 4). There was also a significant difference in the lumen diameter:wall thickness ratio between group 1 and group 2 vessels (Mann–Whitney test, *p* = 0.000; Figure 5A). However, we found a substantial overlap in ranges between groups and were unable to distinguish between any vessels below the average lumen diameter:wall thickness ratio seen in group 2 vessels (4.97, Mann–Whitney test, *p* = 0.817; Figure 5B).

## Discussion

Early research into the ultrastructure of intracerebral blood vessels was hindered by issues with the correct identification of vessel types (Pfeiffer, 1930; Scharrer, 1940; Roggendorf et al., 1978; Roggendorf and Cervós-Navarro, 1983). These studies were based on a classification system established using dermal veins in rabbits (Rhodin, 1968). However, the ultrastructure of venules differs considerably across different organs and thus a criterion based on dermal veins cannot be used as a means of classification in the brain (Rhodin, 1968; Duvernoy et al., 1981; Roggendorf and Cervós-Navarro, 1983). Duvernoy et al. (1981) used resin casts to trace the pathway of intracortical vessels to the large surface pial vessels which were easier to identify. Even this seminal study was not able to clearly differentiate between intraparenchymal vessels concluding that this was difficult using a single plane of section (Duvernoy et al., 1981). Here, we describe a method that utilizes TEM to produce a robust criterion to differentiate parenchymal vessels based on lumen:wall area ratio. It is possible that this criterion may then be applied to other imaging techniques such as standard light microscopy but further work would be required to confirm this.

In the brain of a large mammal (beagle dog), we were able to differentiate parenchymal vessels into two groups based on the widely accepted criteria related to intramural cell morphology and distribution (Rhodin, 1962, 1967, 1968, 1980; Dahl, 1973; Motta, 1990; Armulik et al., 2011). We found that these two groups could be separated by differences in lumen:wall area ratio far more effectively than by using lumen diameter:vessel wall thickness which did not allow for the differentiation of venules that were a similar size to arterioles. As far as we are aware, combining intramural cell morphology with lumen:wall area ratio has not been done before. We found that vessels with a lumen:wall area ratio from 1.89 to 10.96 have discontinuous spindle-like IMC that in the literature have been applicable to venules. However, the larger range of lumen:wall area ratio in these vessels suggests that some ascending venules may have been included. Conversely, we found that vessels with a lumen:wall area ratio from 0.27 to 1.57 have continuous block-like IMC that also in the literature have been applicable to arterioles. We, therefore, conclude that the vessels that we have classified into group 1 are venules and group 2 are arterioles.

Emerging studies have highlighted the previously unrecognized role of venules (Moody et al., 1995, 1997; Brown et al., 2002; Pantoni, 2010; Joutel and Faraci, 2014; Kalaria, 2016) in vascular pathology such as small vessel disease. This has exposed the absence of robust criteria for accurately differentiating between small intracerebral arterioles and venules. In this study, we have shown that intracerebral venules can be successfully differentiated from intracerebral arterioles by simple vessel lumen:wall area ratios. The significance of this is wide and one application is to better understand the types of vessels affected in small vessel disease. The major pathological features of small vessel disease are the changes in small arteries and arterioles in white and gray matter that include arteriolar hyalinosis (collagenosis/fibrosis), fibrinoid necrosis and venous collagenosis. Such changes result in loss of elasticity in the vessel walls and disruption of vessel functions. A reliable system for differentiating between the types of vessels forms the basis for improving the understanding of the pathogenesis and identifying suitable therapeutic targets.

This study was limited to one beagle dog brain and only included vessels that were in transverse section. However, the methods described here could possibly be used to incorporate vessels in obliques section, assuming that all parts of the vessel wall and the lumen are affected by the same degree of distortion caused by oblique sectioning. Similarly, further work is required to ascertain if our method can be utilized in other species.

## Data Availability Statement

The datasets generated for this study are available on request to the corresponding author.

## Ethics Statement

The animal study was reviewed and approved by the local Animal Care Committee and the work was carried out at a facility licensed under the Ontario Animals for Research Act and accredited with the Canadian Council on Animal Care.

## Author Contributions

Electron microscopy and image analysis was performed by MM and TC. HD, CF and AV provided perfused tissue. RC designed the project. All authors contributed equally to the preparation of the manuscript.

## Conflict of Interest

The authors declare that the research was conducted in the absence of any commercial or financial relationships that could be construed as a potential conflict of interest.

## Abbreviations

SMC: smooth muscle cells
IMC: intramural cells
TEM: transmission electron microscopy.
